# A Radical Operation for Procidentia

**Published:** 1865-05

**Authors:** 


A RADICAL OPERATION FOR PROCIDENTIA, read before the New York Obstetrical Society, Dec. 20th, 1864. By THOS. ADDIS EMMET, M.D., Surgeon to the State Woman's Hospital, New York.

This is a neatly printed pamphlet of 17 pages, illustrated by two well-executed cuts. No abstract can be given which would convey an adequate idea of the operation proposed and practiced by Dr. Emmet. We would republish the whole pamphlet in our columns, if the cuts could go with it. But without them it would be so incomplete that we must refer our readers to the pamphlet itself.

